# Post-Learning Offline Pauses Support Consolidation Beyond the Mind-Wandering State

**DOI:** 10.3390/clockssleep8020020

**Published:** 2026-04-17

**Authors:** José Costa Dias, Philippe Peigneux

**Affiliations:** UR2NF—Neuropsychology and Functional Neuroimaging Research Unit, CRCN—Centre for Research in Cognition and Neurosciences, UNI—ULB Neuroscience Institute, Université Libre de Bruxelles (ULB), 1050 Bruxelles, Belgium

**Keywords:** wakeful rest, mind-wandering, local sleep, consolidation, paired-associate learning, offline processing, attentional disengagement

## Abstract

Brief post-learning wakeful resting periods and local sleep mechanisms have been proposed to support offline memory consolidation processes. Mind-wandering (MW), thought to reflect the occurrence or need for local sleep, has been linked to momentary attentional disengagement and may index transitions toward offline processing states. We hypothesized that resting opportunities administered immediately after probe-caught MW episodes reflecting local sleep need may selectively enhance memory consolidation. In a first experiment, participants learned five blocks of eight paired-associate words; a MW thought probe was administered after each block. In the MW condition, participants were allowed a 3 min quiet, offline pause after the block if they reported MW. In the control condition, no pause was administered. Consolidation was better in the MW than the control condition, supporting the hypothesis. However, Experiment 2 tested the MW-related pause effect by comparing the MW condition to a condition in which pauses were allowed irrespective of MW. Results showed that performance equally improved in both conditions, suggesting that post-learning pause effects would not be MW-specific. However, additional analyses evidenced a positive relationship between MW intensity and memory consolidation in both experiments. Our findings suggest that transient interruption of input during a declarative learning session may favor memory consolidation at wake, partially independently of the attentional state.

## 1. Introduction

After an actual learning episode, newly encoded information is gradually transferred into long-term memory. This consolidation process allows recently acquired memory traces to be stabilized and/or reinforced [1,2,3,4]. Sleep is known to contribute offline consolidation [5,6]. In particular, slow-wave sleep (SWS) plays a critical role in the consolidation of hippocampus-dependent spatial and declarative memories [3,7,8]. SWS provides a period of minimal external interference, during which the neurophysiological mechanisms coordinating slow oscillations, spindles, and ripples favor the spontaneous reactivation of memory traces in this offline state [3,7,9,10,11,12].

There are also conditions during wakefulness when cognitive input is minimized and the brain, at least partially, enters an offline mode. Brief periods of quiet wakeful rest following learning have been shown to enhance ulterior memory performance, presumably by reducing ongoing learning practice interference, allowing offline processing mechanisms to develop [13,14,15]. Neural activity observed during wakeful rest has been suggested to resemble some aspects of sleep-like reactivation [16,17], and may thus benefit memory consolidation. However, findings are inconsistent, with some studies reporting strong benefits and others no measurable effects [18,19,20,21,22]. These discrepancies suggest that rest may support consolidation under specific conditions only, highlighting the need to determine not only whether those pauses are beneficial, but also when they are most effective in the learning process. On these premises, it can be hypothesized that the effectiveness of offline pauses depends on the time point at which the learner disengages from encoding. During prolonged wakefulness, increasing sleep pressure leads to reduced alertness and a decline in performance [23,24,25], a phenomenon explained in the framework of the Borbély’s Process S component of sleep regulation, in which accumulation of sleep pressure corresponds to a rising homeostatic load that increases the likelihood of slow-wave expression during sleep [23]. Expanding to sustained cognitive activity, theoretical accounts proposed that local neural fatigue within task-relevant networks may trigger transient offline states during wakefulness, a phenomenon referred as local sleep [26,27,28,29]. Local sleep was associated with slow-wave activity (SWA) in the delta range (0.5–4 Hz) and reduced behavioral performance [27,30,31,32]. During post-training sleep, SWA locally increases in brain regions engaged during learning, and greater SWA was found to predict enhanced memory performance the following day [6,31].

**Figure 1:**
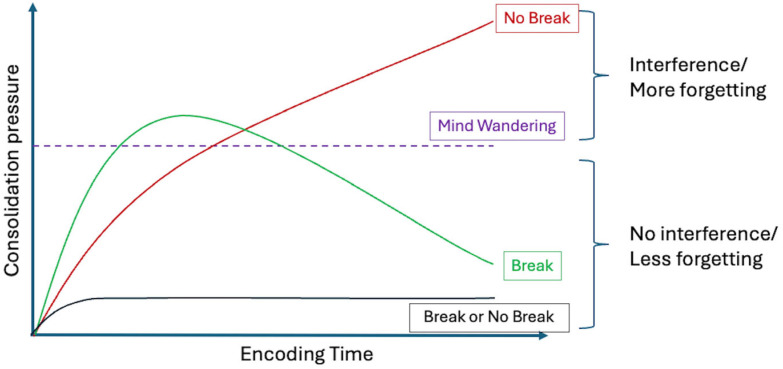
Theoretical consolidation pressure framework. As encoding continues, consolidation load accumulates (increased consolidation pressure). When it exceeds a given threshold (purple dashed line), transient offline disengagement and MW emerge. If no break is taken (red line), the consolidation pressure continues rising, impairing performance and stabilization of memory traces. If a break is allowed shortly after disengagement (green line), then consolidation pressure drops, preserving learning capabilities and favoring offline memory processing. The lower black curve illustrates a focused encoding condition in which consolidation pressure always remains below threshold.

Although elevated SWA may be considered a neural state that favors consolidation, its presence during wakefulness is usually viewed as behaviorally detrimental, being associated with attentional lapses, slower responses, and increased error rates [27,28,29,30,32,33,34]. Local build-up of homeostatic sleep pressure has also been proposed a key mechanism in triggering mind-wandering (MW) [27,35], defined as a shift in attention away from the ongoing task toward internally directed thought. Accordingly, the occurrence of MW was correlated to attentional lapses, increased error rates and SWA [36,37,38,39]. In this respect, MW can also be viewed as a correlate of local sleep-like need and promotes an offline learning mode that would be beneficial for memory consolidation mechanisms. Hence, MW may reflect moments when local sleep need increases and encoding efficiency declines. Building on this framework, we hypothesized that sustained encoding would generate increasing consolidation demands (in other words, a consolidation pressure) such that continuing to input new information would eventually become less efficient than briefly disengaging (see Figure 1). In a nutshell, we propose that a transient offline disengagement state emerges when consolidation pressure increases over a specific threshold. If, at this point, learning continues, consolidation pressure will continue rising and impair performance. If, on the contrary, a break is provided when disengagement manifests (e.g., through increased MW), it will relieve consolidation pressure, both restoring learning capabilities and allowing memory consolidation mechanisms to unfold during the break.

**Figure 2:**
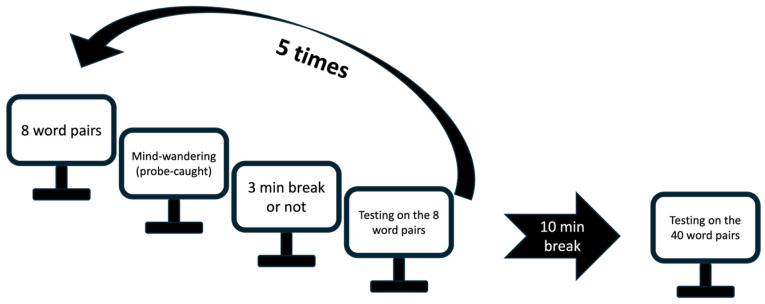
Experimental design Study 1. Participants learned 5 blocks of 8 word pairs. After each block, a probe-caught mind-wandering question assessed their attentional state. In blocks 2, 3 or 4 in the MW condition, a 3 min quiet resting break was administered immediately after the probe if participants reported mind-wandering. In the control condition, no pause was allowed irrespective of the output of the MW probe. Participants completed after each block (and pause if any) an immediate cued-recall test on the same 8 word pairs with corrective feedback. The sequence was repeated five times for a total of 40 learned word pairs. In both conditions, participants had a 10 min break after block 5, followed by a final cued-recall test on the 40 learned word pairs, without feedback. Participants were successively administered both conditions in a randomized order with a 10 min interval between conditions filled in with a psychomotor vigilance task.

To test this hypothesis, we used a paired-associate learning paradigm with probe-caught MW thoughts sampling (Figure 2) across two successive experiments. In Experiment 1, we tested whether pauses introduced immediately after MW reports during the learning phase improved memory at delayed recall as compared to a continued encoding condition. Experiment 2 tested whether MW-associated pause benefits were genuinely specific to MW by comparing pauses following MW versus pauses following focused-attention episodes (see Section 4 for methodological details).

## 2. Results

### 2.1. Study 1

We tested in a final sample of 34 young healthy participants who were administered both experimental conditions, whether introducing a pause immediately after a probe-caught mind-wandering (MW) report would improve delayed recall as compared to the continued learning condition (see Section 4.1.1 Study 1). Memory consolidation was quantified computing a consolidation index (i.e., delayed recall total score minus immediate recall total score).

#### 2.1.1. Break Following Mind-Wandering vs. Continued Learning

**Figure 3:**
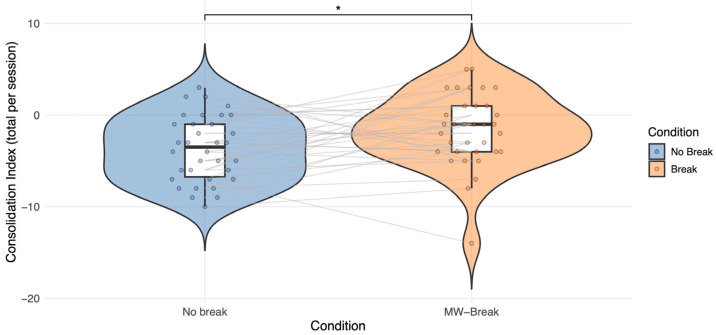
Study 1. Individual participant’s consolidation scores in the No-Break and MW-Break conditions are displayed in gray, illustrating within-subject variability. Colored dots represent raw consolidation index scores for each condition and participant. A violin plot containing boxplots summarize the distribution within each condition. A significant Condition effect is observed, with a higher consolidation score (i.e., less forgetting) in the MW-Break than in the No-Break (*p* = 0.016 [*]) condition.

To assess whether a break following MW enhances memory consolidation, data were analyzed using a linear mixed-effects model with the experimental Condition (No-Break vs. MW-Break) as the predictor of interest (see Section 4.3 Model Specification). The analysis was computed on 68 observations (N 34 × 2 Conditions). The model revealed a significant Condition effect on the consolidation index (*β* = 2.24, *SE* = 0.88, *t*(31) = 2.56, *p* = 0.016, 95% CI [0.52, 3.96]) with a higher consolidation score when a pause was administered immediately after a MW report (Figure 3). To ensure that the observed effect of the Condition was not driven by model specification, we conducted two complementary analyses. First, we fitted a reduced model including Condition only, without covariates, which yielded an effect of Condition comparable in direction and magnitude to that observed in the full model (Appendix A.1; Table A1). Second, we performed a likelihood ratio test comparing the full model with an otherwise identical model excluding Condition. This analysis showed that the inclusion of Condition significantly improved model fit (*χ*^2^ (1) = 6.85, *p* = 0.009; see Appendix A.2; Table A2). Additional within-condition analyses indicated that variability in the number of pauses within the MW-Break condition did not significantly predict consolidation performance (*p* = 0.58; see Appendix A.3; Table A3). Bayesian linear mixed-effects analyses yielded results consistent with the frequentist approach, showing a positive effect of *Condition* on the consolidation index (*β* = 2.17, 95% CrI [0.5, 3.86]). The Bayes factor provided positive evidence in favor of the alternative hypothesis relative to the null hypothesis of no effect (BF_10_ ≈ 4.08), using weakly informative priors. For descriptive purposes, we also examined the within-subject correlation of the consolidation index across conditions. The correlation between the No-Break and MW-Break conditions was low (r = 0.16).

#### 2.1.2. Sensitivity and Power Analysis

To further characterize the evidential value of Study 1, we conducted a sensitivity power analysis on the within-subject difference in consolidation index (MW-Break vs. No-Break). With a final sample of *n* = 34 participants, the study had 62% power to detect a moderate effect size (*dz* = 0.40, two-tailed *α* = 0.05), corresponding to a beta-error probability of 38%. The minimum detectable effect size with 80% power was *dz* = 0.50.

#### 2.1.3. Moderation by Mind-Wandering Intensity

**Figure 4:**
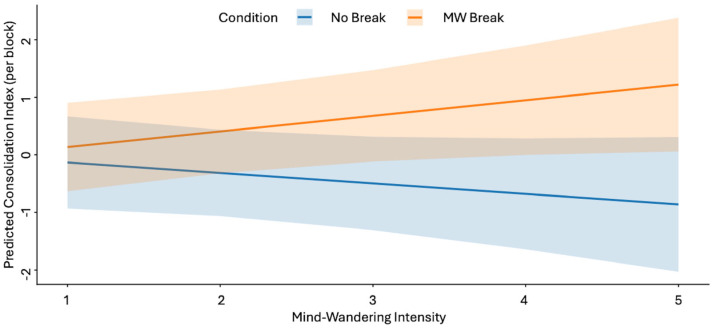
Study 1. Model-estimated marginal means of the consolidation index across levels of mind-wandering intensity for the No-Break and MW-Break conditions. Shaded areas indicate 95% confidence intervals. Predictions are derived from a linear mixed-effects model fitted to blocks 2–4 where the interaction between Condition and MW Intensity is significative (*p* = 0.02). MW intensity: 1 = focused, 2 = mostly focused, 3 = intermediate, 4 = mostly distracted, 5 = highly distracted.

To test whether mind-wandering intensity moderated the effect of experimental Condition, we restricted the analysis to the blocks in which breaks were implemented (blocks 2–4) and fitted a linear mixed-effects model with the interaction between Condition (No-Break vs. MW-Break) and mind-wandering intensity (1 = focused, 2 = mostly focused, 3 = intermediate, 4 = mostly distracted, 5 = highly distracted). Mind-wandering intensity was treated as a continuous variable, based on the hypothesis that it reflects the proportion of local sleep need. To be able to assess the impact of the mind-wandering, we need to do an analysis based on individual block outcomes. Thus, for each block where the condition could be triggered, the consolidation index was computed as the difference between delayed and immediate recall scores within the same block (e.g., delayed recall for word pairs in block 3 minus immediate recall in block 3). The analysis was computed on 204 observations (N 34 × 3 blocks × 2 conditions). The model highlighted a significant effect of the interaction Condition × Intensity on the consolidation index (*β* = 0.45, *SE* = 0.19, *t*(188) = 2.35, *p* = 0.02, 95% CI [0.07, 0.83]); indicating that the beneficial effect of MW-Breaks increased with higher levels of mind-wandering (Figure 4).

Consistent with the interaction reported above, exploratory post hoc contrasts based on estimated marginal means indicated that the benefit of MW-triggered breaks increased with higher levels of mind-wandering intensity. No reliable difference between MW-Break and No-Break Conditions was observed at the lowest level of mind-wandering, whereas progressively larger benefits of MW-Breaks emerged at intermediate and high levels of mind-wandering (Appendix B, Table A4).

#### 2.1.4. Interim Discussion

Study 1 results showed that allowing a pause immediately after a MW report improves delayed memory recall, which was impacted by the intensity of the mind-wandering. These results are seemingly on agreement with our hypothesis that providing a short quiet rest opportunity when disengagement manifests through a MW report would relieve the accumulated pressure, both restoring learning capabilities and allowing memory consolidation mechanisms to unfold. However, a limitation of the experimental design of Study 1, directly comparing a MW-related break condition to a continued encoding with No-Break condition, is that it does not allow to disentangle whether the observed memory consolidation benefit in the MW-related break condition is specifically due to the occurrence of MW (and putatively local sleep) or merely to the quiet rest opportunity and decreased external input irrespective of the participant’s attentional state, i.e., mind-wandering or focused on the task. To test this alternative possibility, we conducted Study 2. The experimental protocol was identical to Study 1 for the mind-wandering (MW) condition, with a pause allowed when participants reported MW after blocks 2, 3 or 4. In the focused-attention (FA) condition, participants were allowed a pause after blocks 2, 3 or 4 when participants reported being focused on the learning task (i.e., not mind-wandering).

### 2.2. Study 2

We tested in a final sample of 19 young healthy participants who were administered both experimental conditions whether introducing a pause immediately after a probe-caught mind-wandering (MW) report would improve delayed recall as compared to a pause allowed after a focused attention report. Memory consolidation was again quantified computing the consolidation index (i.e., delayed recall total score minus immediate recall total score).

#### 2.2.1. MW-Break vs. FA-Break

**Figure 5:**
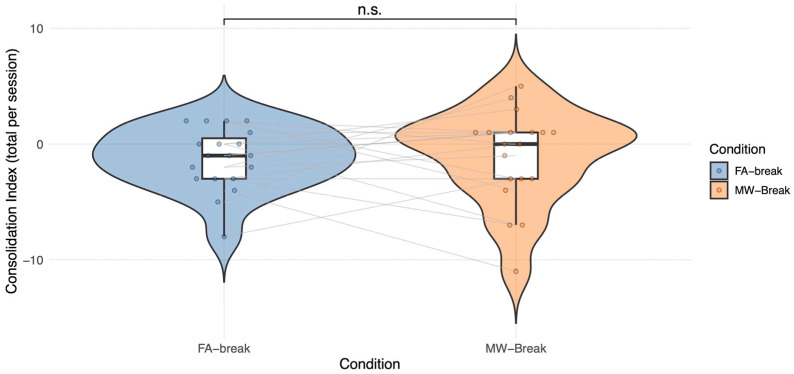
Study 2. Individual participant’s scores in the FA-Break and MW-Break Conditions are displayed in gray, illustrating within-subject variability. Colored dots represent raw consolidation index scores for each Condition and participant. A violin plot containing boxplots summarize the distribution within each Condition. No significant Condition effect is observed (*p* = 0.85; n.s. = non-significant).

We used a linear mixed-effects model on 38 observations (N 19 × 2 sessions) to test whether break condition (FA-Break vs MW-Break) influenced the consolidation index, calculated as in Study 1. The model revealed no significant Condition effect on the consolidation index (*β* = 0.17, *SE* = 0.91, *t*(16) = 0.19, *p* = 0.85, 95% CI [−1.61, 1.95], see Figure 5). To ensure that the observed effect of the condition was not driven by model specification, we conducted two complementary analyses as in Study 1. The reduced model including Condition only, yielded a non-significant effect of Condition comparable in direction and magnitude to that observed in the full model (Appendix C.1; Table A5). Second, we performed a likelihood ratio test comparing the full model with an otherwise identical model excluding Condition. As expected, this analysis showed that the inclusion of Condition did not significantly improve model fit (*χ*^2^ (1) = 0.04, *p* = 0.84; see Appendix C.2; Table A6). Including the number of breaks as a covariate revealed a modest positive effect of the number of breaks on consolidation index (*p* = 0.05) but did not change the non-significance of the Condition effect (*p* = 0.77). Also, the number of breaks did not differ between FA and MW conditions (*p* = 0.86) (Appendix C.3; Table A7).

Bayesian linear mixed-effects analyses yielded results consistent with the frequentist approach, showing no effect of Condition on the consolidation index (β = 0.17, 95% CrI [−1.71, 2.06]). The estimated Bayes factor indicated positive evidence for the null hypothesis (BF_10_ ≈ 0.20; BF_01_ ≈ 5.25). The within-subject correlation of the consolidation index between the FA-Break and MW-Break conditions was low (*r* = 0.14).

#### 2.2.2. Sensitivity and Power Analysis

With a final sample of *n* = 19 participants, the study had 38% power to detect a moderate effect size (*dz* = 0.40, two-tailed *α* = 0.05), corresponding to a beta-error probability of 62%. The minimum detectable effect size with 80% power was *dz* = 0.68. The observed effect size was negligible (*dz* = 0.03). Accordingly, the present data allow us to exclude large effects (*dz* ≥ 0.68), whereas small-to-moderate effects cannot be formally ruled out.

#### 2.2.3. Condition × MW Intensity

**Figure 6:**
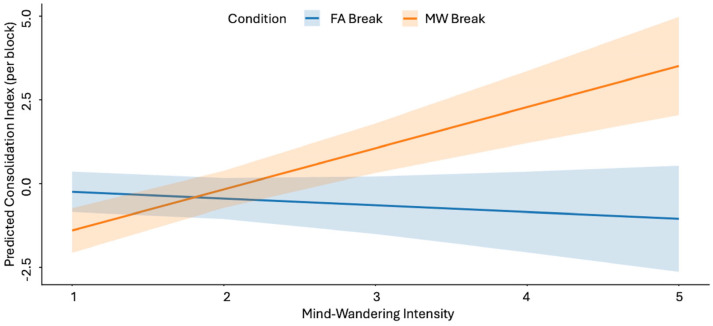
Study 2. Model-estimated marginal means of the consolidation index across levels of mind-wandering intensity for the FA-Break and MW-Break conditions. Shaded areas indicate 95% confidence intervals. Predictions are derived from a linear mixed-effects model fitted to blocks 2–4 where the interaction between Condition and MW Intensity is significative (*p* < 0.001). MW intensity: 1 = focused, 2 = mostly focused, 3 = intermediate, 4 = mostly distracted, 5 = highly distracted.

To test whether mind-wandering intensity moderated the effect of experimental condition, we used the same procedure as in Study 1. A linear mixed-effects model on 114 observation (N 19 × 3 Blocks × 2 sessions) revealed a significant interaction between break Condition and mind-wandering intensity ((*β* = 1.42, *SE* = 0.29, *t*(100) = 4.88, *p* < 0.001, 95% CI [0.85, 2]). Specifically, the effect of MW-triggered breaks on the consolidation index depended on the level of reported mind-wandering (Figure 6).

Exploratory post hoc contrasts based on estimated marginal means indicated that MW-triggered breaks were associated with lower consolidation scores at the lowest level of mind-wandering, no reliable difference at intermediate levels, and progressively higher consolidation scores at higher levels of mind-wandering (Appendix D; Table A8).

### 2.3. Encoding Performance and Delayed Recall

Since post-learning pauses may influence memory either by facilitating consolidation or improving encoding efficiency, we assessed immediate recall performance and its contribution to delayed retrieval outcomes in both Study 1 and Study 2.

#### 2.3.1. Study 1

To determine whether break condition influenced encoding performance, we first examined immediate recall scores as an index of encoding strength. Immediate recall data were analyzed using a linear mixed-effects model including Condition (No-Break vs. MW-Break) as predictor. The analysis was computed on the 68 observations (N 34 × 2 Conditions) used in the main analysis. The analysis revealed no significant Condition effect on immediate recall performance (*β* = 0.61, *SE* = 1.00, *t*(31) = 0.6, *p* = 0.55, 95% CI [−1.36, 2.57]), indicating comparable encoding performance across the two experimental conditions.

**Figure 7:**
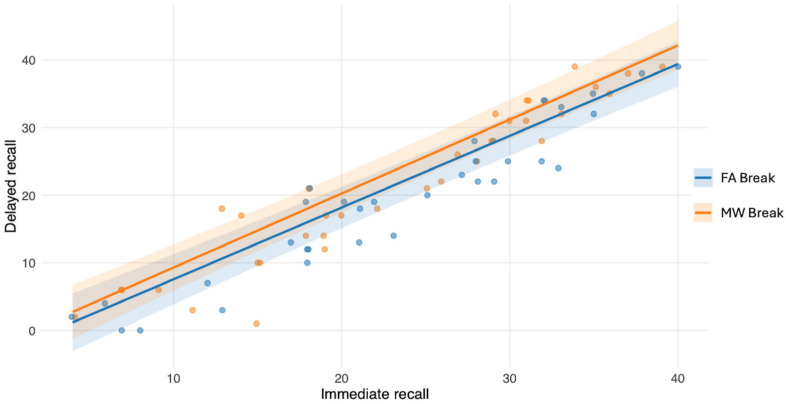
Study 1. Relationship between immediate recall (encoding strength) and delayed recall performance. Each point represents one session (No-Break vs. MW-Break). Lines represent model-estimated predictions derived from the linear mixed-effects model, with shaded areas indicating 95% confidence intervals. Delayed recall increased with encoding strength, while slopes did not differ between conditions, consistent with the absence of an interaction effect.

We next tested whether encoding strength predicted delayed memory retrieval after the 10 min delay, and whether this relationship differed between conditions. A linear mixed-effects model was fitted with delayed recall as the dependent variable and immediate recall (mean-centered to facilitate interpretation of main effects), Condition, and their interaction as predictors. The analysis was computed on the 68 observations (N 34 × 2 Conditions) used in the main analysis. Immediate recall significantly predicted delayed recall (*β* = 1.06, *SE* = 0.07, *t*(59) = 15.8, *p* < 0.01, 95% CI [0.93, 1.19]), indicating that better encoding was associated with improved later retrieval. The main Condition effect was significant (*β* = 2.18, *SE* = 0.88, *t*(59) = 2.48, *p* = 0.02, 95% CI [0.46, 3.91]). Critically, the Immediate recall × Condition interaction was not significant (*β* = 0.03, *SE* = 0.09, *t*(59) = 0.35, *p* = 0.73, 95% CI [−0.15, 0.22]), suggesting that the relationship between encoding strength and delayed retrieval did not differ between break conditions (Figure 7).

#### 2.3.2. Study 2

Immediate recall data were analyzed using a linear mixed-effects model including the predictor Condition (FA-Break vs. MW-Break) as predictor. The analysis was computed on the 38 observations (N 19 × 2 sessions) used in the main analysis. The analysis revealed no significant Condition effect on immediate recall performance (*β* = 0.63, *SE* = 1.26, *t*(16) = 0.5, *p* = 0.63, 95% CI [−1.84, 3.09), indicating comparable encoding performance across experimental conditions.

**Figure 8:**
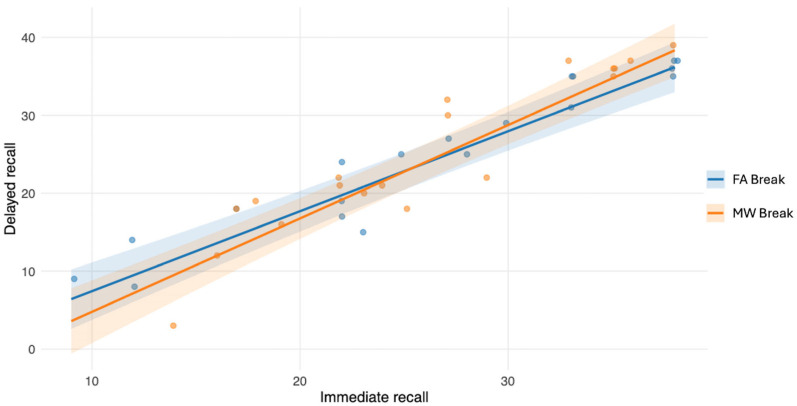
Study 2. Relationship between immediate recall (encoding strength) and delayed recall performance. Each point represents one experimental session (FA- vs. MW-Break). Lines represent model-estimated predictions derived from the linear mixed-effects model, with shaded areas indicating 95% confidence intervals. Delayed recall increased as a function of encoding strength, while regression slopes were comparable across conditions, consistent with the absence of an interaction between encoding level and break condition.

We next tested whether encoding strength predicted delayed memory retrieval after the 10 min delay, and whether this relationship differed between conditions. A linear mixed-effects model was fitted with delayed recall as the dependent variable and immediate recall (mean-centered to facilitate interpretation of main effects), Condition, and their interaction as predictors. The analysis was computed on the 38 observations (N 19 × 2 sessions) used in the main analysis. Immediate recall significantly predicted delayed recall (*β* = 1.03, *SE* = 0.09, *t*(31) = 11.87, *p* < 0.01, 95% CI [0.86, 1.2]), indicating that better encoding was associated with improved later retrieval. The main Condition effect was not significant (*β* = 0.14, *SE* = 0.89, *t*(14) = 0.15, *p* = 0.88, 95% CI [−1.61, 1.88]). Critically, the Immediate recall × Condition interaction was not significant (*β* = 0.17, *SE* = 0.12, *t*(17) = 1.4, *p* = 0.18, 95% CI [−0.07, 0.41]), suggesting that the relationship between encoding strength and delayed retrieval did not differ between break conditions (Figure 8).

### 2.4. Exploratory Analysis

#### 2.4.1. The Effect of Break (Study 1 + Study 2)

**Figure 9:**
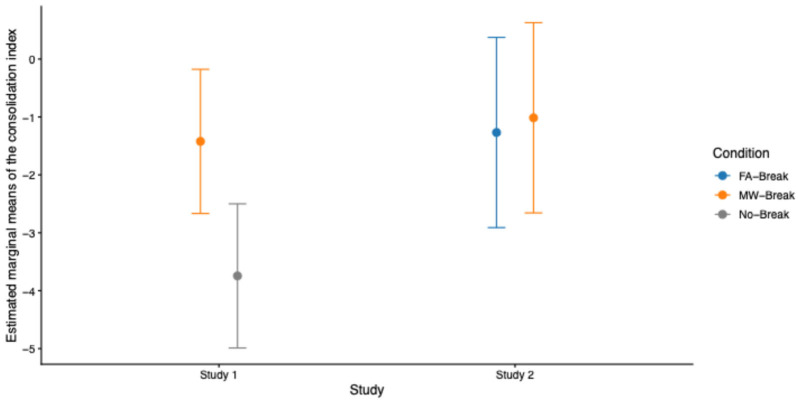
Estimated marginal means of the consolidation index from the linear mixed-effects model for combining Study 1 and Study 2. Error bars represent 95% confidence intervals. In Study 1, consolidation is lower in the No-Break condition relative to the MW-Break condition, whereas in Study 2, consolidation does not differ between MW-Break and FA-Break conditions.

To further assess whether post-learning breaks facilitate memory consolidation and under which conditions, we conducted planned contrast analyses on Study 1 and Study 2 combined. First, we tested the interaction effect between studies (Study 1 vs. Study 2) and break conditions using a linear mixed-effects model fitted to 106 observations (53 participants ((N = 34 for Study 1, N = 19 for Study 2) × 2 sessions). The consolidation index was computed in both individual studies. The model revealed no significant interaction between study and break condition on the consolidation index (*β* = −2.07, *SE* = 1.34, *t*(49) = −1.54, *p* = 0.13, 95% CI [−4.70, 0.56]), indicating that the effect of breaks did not differ reliably between studies. Post hoc planned contrasts analyses based on estimated marginal means were conducted to test specific comparisons of interest. These analyses showed that the No-Break condition in Study 1 was associated with significantly lower consolidation compared to the MW-Break condition in Study 2 (*β* = 2.73, *SE* = 1.05, *t*(97) = 2.6, *p* = 0.01) and to the FA-Break condition in Study 2 (*β* = 2.48, *SE* = 1.04, *t*(96) = 2.38, *p* = 0.02). At variance, consolidation following MW-Breaks in Study 1 did not differ from either FA-Breaks (*β* = −0.15, *SE* = 1.05, *t*(97) = −0.15, *p* = 0.88) or MW-Breaks (*β* = −0.41, *SE* = 1.04, *t*(96) = −0.39, *p* = 0.7) in Study 2 (Figure 9), showing that improved memory consolidation scores are mostly related to the presence of breaks after learning blocks, irrespective of MW or FA reports (but see Condition × MW intensity results in Study 1 and Study 2).

#### 2.4.2. Vigilance and Time-of-Day Factors

Because the present study focuses on attentional engagement during learning, we conducted exploratory analyses to examine whether vigilance and circadian timing factors could account for variability in performance or pause-related effects in Study 1 and Study 2.

First, we tested whether vigilance differed as a function of the condition order (i.e., the condition completed first). Vigilance was indexed using reciprocal reaction time (RRT), calculated as the inverse of reaction time (1/RT) during the Psychomotor Vigilance Task (PVT). This metric was chosen because it provides superior statistical properties by reducing the disproportionate influence of extremely long reaction times [40]. In Study 1, a two-sample t-test comparing RRT performance between participants who started with the MW-Break condition versus the No-Break condition revealed no reliable difference (*t*(32) = −0.69, *p* = 0.5, 95% CI [−0.26; 0.13]). Likewise, in Study 2, the same analysis comparing participants who started with the MW-Break versus the FA-Break condition revealed no statistically significant difference (*t*(33) = −1.47 *p* = 0.16, IC 95% [−0.46; 0.08]). To assess whether vigilance differed between studies, we conducted a two-way between-subjects ANOVA on RRT with Study and Condition order as factors. This analysis revealed no significant main effects of Study or Condition order, and no Study × Condition order interaction (study: *F*(1,49) = 0.36, *p* = 0.55; condition order: *F*(1,49) = 1.79, *p* = 0.19; interaction: *F*(1,49) = 0.53, *p* = 0.47).

Second, we assessed whether the two samples differed in chronotype. A two-sample t-test comparing the Morningness–Eveningness Questionnaire [41] scores between Study 1 and Study 2 indicated that chronotype did not reliably differ across Studies (*t*(42) = −1.46, *p*= 0.15, 95% CI [−10.65; 1.69]).

Third, we examined whether the effect of break condition varied as a function of the alignment between the time of testing and the participants’ subjective time-of-day preferences (i.e., when they reported being most versus least efficient). Participants indicated their subjectively optimal and least optimal time of day by selecting one of six 4 h time windows (01:00–04:00, 05:00–08:00, 09:00–12:00, 13:00–16:00, 17:00–20:00, 21:00–24:00). The time of testing was assigned to the corresponding window. Alignment was operationalized as the circular distance (ranging from 0 to 3) between the testing window and the selected time-of-day window, such that the last and first windows were treated as adjacent (e.g., 21:00–24:00 and 01:00–04:00). Separate alignment measures were computed for the subjectively reported best and worst time-of-day windows. We then tested whether alignment moderated the effect of break condition on the consolidation index by adding an interaction term (Condition × Alignment) to the linear mixed-effects model. Alignment with the subjectively reported best time of day did not reliably moderate the effect of Break Condition on the consolidation index (Study 1: *β* = 1.77, *SE* = 1.36, *t*(30) = 1.3, *p* = 0.2, 95% CI [−0.89, 4.43]; Study 2: *β* = −2.11, *SE* = 1.46, *t*(15) = −0.45, *p* = 0.17, 95% CI [−5, 0.74]), as is similar with the subjectively reported worst time of day (Study 1: *β* = −1.42, *SE* = 1.14, *t*(30) = −1.24, *p* = 0.22, 95% CI [−3.65, 0.82]; Study 2: *β* = 0.2, *SE* = 0.94, *t*(15) = 0.21, *p* = 0.83, 95% CI [−1.64, 2.05]).

## 3. Discussion

### 3.1. Interpretation of Empirical Findings

Across two experiments, we examined whether brief pauses inserted immediately after internally reported attentional disengagement (i.e., mind-wandering [MW]) selectively benefit declarative memory consolidation for verbal material. Although Study 1 showed evidence for improved delayed recall performance and memory consolidation when a pause was allowed during the learning session after a mind-wandering (MW) report, Study 2 demonstrated that this advantage was not specific to the MW state in itself since performance equally improved when pauses were allowed either after a mind-wandering or after a focused attention report. Notwithstanding, intensity analyses suggested that the effect of pauses may vary with MW intensity; the higher the MW intensity, the higher the consolidation index. Thus, our results do not entirely support our initial assumption that MW reports would signal a privileged window for disengagement, linked to the development of local sleep needs. Instead, it mostly suggests an unspecific beneficial effect of short quiet resting breaks during the learning process, beyond mind-wandering effects.

Importantly, additional analyses examining encoding performance indicated that the observed memory effects could not be explained by differences in initial learning levels. In both studies, immediate recall performance did not differ between break conditions, suggesting comparable encoding efficiency. Although encoding strength strongly predicted delayed recall, it did not interact with the break condition (either in Study 1 No-Break vs. MW-Break, or in Study 2 FA- vs. MW-Break), indicating that the relationship between encoding and later retrieval remained stable across experimental manipulations. These findings therefore support the interpretation that post-learning pauses primarily influence offline memory processes rather than encoding itself.

Our results contribute to the ongoing debate about offline processing mechanisms taking place during wakefulness. Prior studies showed that wakeful rest can protect newly acquired traces from interference [12,13,14], yet mixed findings have raised questions regarding the boundary conditions of such benefits [18,19,20,21]. The present data suggest that the main critical factor may not be the internal state triggering the pause. Rather, the cessation of ongoing input due to presentation of the learning material may be sufficient to support memory consolidation mechanisms. Although MW has been associated with behavioral lapses and reduced cortical responsiveness in other paradigms [33,34,35,36], the present findings do not allow us to infer a mechanistic link between attentional drift, putative offline states, and consolidation. Moreover, MW is a complex and multimodal phenomena [42,43,44], and self-reported attentional states may capture heterogeneous underlying processes.

The present findings may also be discussed in relation to the literature on massed versus spaced learning. The insertion of brief pauses during encoding may share functional similarities with spacing effects, i.e., improved long-term retention performance when learning episodes are distributed over time [45,46]. From this perspective, the pauses we introduced could have temporarily reduced ongoing encoding demands and allowed partial stabilization or refreshing of recently encoded representations, even if spacing effects are usually tested with longer intervals than the 3 min breaks in the present study. Also, this interpretation remains tentative since pause duration was not experimentally controlled here. Beyond long-term memory consolidation accounts, the present findings may also be discussed considering time-based models of working memory, such as the Time-Based Resource-Sharing (TBRS) framework [47]. According to the TBRS model, processing and maintenance compete for limited attentional resources, such that memory traces decay when attention is continuously engaged, but can be refreshed when brief temporal windows become available. Although our study targeted long-term memory consolidation rather than working memory maintenance, a conceptual parallel may exist whereby pauses during encoding temporarily reduce processing demands, potentially allowing stabilization processes to occur. These similarities should nevertheless be interpreted cautiously, as the present paradigm was not designed to directly test working memory mechanisms. Future studies are needed to systematically test these potentially contributing factors.

### 3.2. A Speculative Theoretical Framework

The following framework is intended as a speculative conceptual interpretation designed to organize the present behavioral findings rather than as a directly tested mechanistic model.

**Figure 10:**
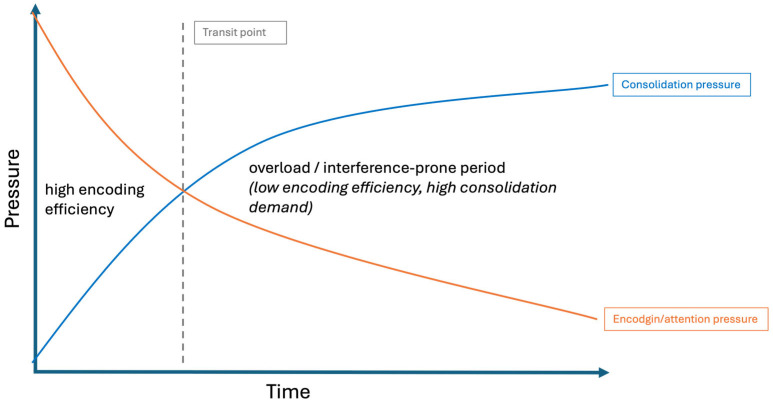
Conceptual schematic of a dual pressure dynamics model. Encoding pressure gradually declines while consolidation pressure increases, potentially creating intervals during which continued input may interfere with stabilization. Note that this transition zone is purely theoretical and not directly inferred from the present behavioral data.

Beyond the general benefit of pauses observed in this experiment, our findings raise the question of how internal cognitive dynamics determine the optimal moment to disengage from encoding. We propose here a hypothetical framework in which two pressures evolve jointly over the course of learning: an attentional (or encoding) pressure, reflecting the system’s ability to maintain task-oriented processing, and a consolidation pressure, corresponding to a growing need to stabilize recently encoded traces (Figure 10).

This dual-dynamics perspective may help with contextualizing variability in pause-related outcomes across studies, suggesting that the efficacy of rest periods during the learning process depends less on its duration than on its temporal alignment with internal processing demands. When consolidation pressure remains low, pauses may confer little measurable benefit; once consolidation demands exceed encoding efficiency, continued input may generate interference, and brief disengagement may reduce it. Under overload conditions, however, even pauses may not be sufficient to prevent decay.

This framework conceptually aligns with the Complementary learning systems account [10] that distinguishes rapid hippocampal encoding from slower neocortical integration that requires interference reduction. It also converges with opportunity-cost approaches to attention and effort in which withdrawal reflects adaptive recalibration rather than breakdown [48]. In this perspective, local sleep may not mark a mechanism of consolidation, but rather a potential reallocation cue under conditions of diminishing encoding return. Although speculative, this proposal resonates with reports of local sleep intrusions following cognitive load [27,32] and with accounts linking attentional decline to consolidation requirements [49]. Crucially, however, the present data do not allow mechanistic inference. While our analyses suggest that the benefit of breaks may vary with the reported intensity of mind-wandering, this factor was not experimentally controlled and should therefore be interpreted with caution. Future research combining real-time MW sampling with neural slow-wave markers, pupillometry, and temporally resolved modeling will be required to test whether offline disengagement and consolidation-related pressures co-evolve dynamically, and whether the effectiveness of brief pauses depends on their timing within broader circadian and cognitive dynamics, potentially opening the way for a chronobiological account of when pauses are most effective.

## 4. Materials and Methods

### 4.1. Participants

All participants gave their written informed consent to participate in those studies approved by the ethics advisory committe of the ULB Faculty of Psychology (agreement # 1760/2024).

#### 4.1.1. Study 1

**Figure A1:**
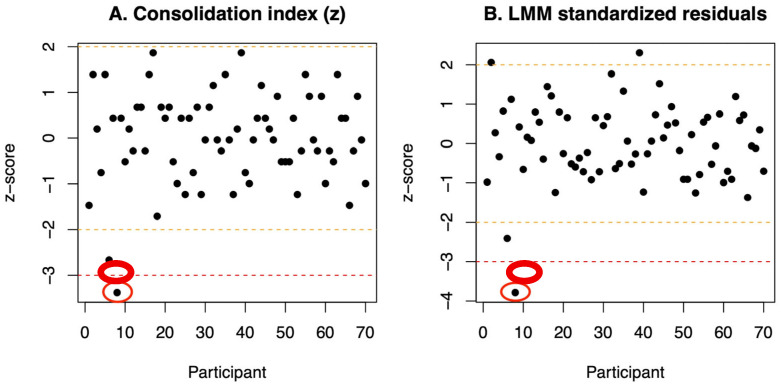
(**A**) Identification of an outlier based on standardized Consolidation index. Consolidation indexes are shown for all participants. Dashed orange lines indicate ±2 standard deviations, and the red dashed line indicates the exclusion threshold at −3 standard deviations. (**B**) Identification of an outlier based on standardized residuals. Standardized residuals from the linear mixed-effects model are shown for all participants. Dashed orange lines indicate ±2 standard deviations, and the red dashed line indicates the exclusion threshold at −3 standard deviations. (**A**,**B**) The circled data point corresponds to the same participant whose residual and consolidation index exceeded the threshold and was therefore classified as an outlier.

A total of 48 healthy young adults participated in Study 1. Participants were recruited through three modalities: the Université libre de Bruxelles (ULB) SONA recruitment platform, social media advertisements, and informal peer dissemination. SONA participants received course credits as compensation, the others participated without any compensation. Inclusion criteria required participants to be between 18 and 30 years of age. Because the aim of the analysis was to test the effect of break occurrence, we excluded participants who did not report MW and were thus not administered a break during the MW-triggered break condition. In addition, participants with a consolidation index exceeding three standard deviations from the mean (|*z*| > 3), based on model residuals, were considered outliers and excluded from the analysis (see Appendix E; Figure A1). The final sample resulted in 34 participants (24 females, 10 males; M age = 23.26 years, SD = 3.65). Educational-level completion was as follows: 12 participants with upper-secondary education, 8 with a bachelor’s degree, 13 with a master’s degree, and 1 with a doctoral-level degree.

#### 4.1.2. Study 2

A total of 40 healthy young adults participated in the study. Participants were recruited through social media advertisements and received 20 € as compensation for their participation. Inclusion/exclusion criteria were the same as Study 1. Only participants who were administered a break in both MW and FA conditions were retained for the final analyses. No participants had an index of consolidation more than three standard deviation from the mean (|z|> 3) and in the residuals of the model, resulting in a final sample of 19 participants (13 females, 5 males, 1 non-binary; M age = 22.89 years, SD = 3.9). Educational-level completion was as follows: 9 participants with upper-secondary education, 4 with a bachelor’s degree, 5 with a master’s degree, and 1 with a doctoral-level degree.

#### 4.1.3. Study 1 + Study 2

Participants used in Study 1 and Study 2 analyses were retained, resulting in 53 participants (34 Study 1 + 19 Study 2; 37 females, 15 males, 1 non-binary; M age = 23.13 years, SD = 3.71).

### 4.2. General Procedure

#### 4.2.1. Questionaries

Prior to the experimental task, participants completed a brief demographic form (age, gender, education level) and exploratory items assessing chronotype and subjective time-of-day performance. Chronotype was assessed using a French version of the Morningness–Eveningness Questionnaire [41]. Subjective time-of-day optimal and non-optimal performance was assessed by asking participants to indicate the time intervals during which they felt most and least cognitively efficient, respectively (1 h to 4 h, 5 h to 8 h, 9 h to 12 h, 13 h to 16 h, 17 h to 20 h, and 21 h to 24 h).

#### 4.2.2. Experimental Designs

The two studies employed a within-subject design with two learning sessions. Each session comprised 40 word pairs presented in five blocks of eight pairs. Word pairs were disyllabic and selected randomly from the BRULEX database, a standardized French lexical resource providing frequency, phonological, and lexical norms (https://crcn.ulb.ac.be/lab_post/brulex-2/ accessed on 2 February 2024). Two equivalent lists were created and counterbalanced across participants. Word pairs were displayed for 5 s each on a computer screen, and participants completed an immediate cued-recall test with feedback after every block (i.e., they were presented the first word of the pair and had to recall the second, associated word).

After each block of eight pairs, a probe-caught attentional rating Likert scale was administered (from 1 = Fully focused on the task (no mind-wandering). 2 = Mostly focused on the task, with occasional background thoughts. 3 = Split between the task and other thoughts. 4 = Mostly disengaged, but still loosely following the learning task. 5 = Completely disengaged, no longer paying attention to the words). In the pause-allowed conditions (see below), a 3 min quiet rest period could be introduced after blocks 2, 3, or 4 (maximum three breaks per session), depending on their attentional state. Study 1: In the MW condition, a rest period was allowed whenever participants reported attentional disengagement (Likert scale score > 1) after blocks 2, 3, or 4. In the No-Break session, no pauses were introduced irrespective of the outcome of the Likert scale. Study 2: Breaks were administrated in two different attentional states in separate sessions. In the MW-Break session, a 3 min rest was introduced after blocks 2, 3, or 4 when disengagement was reported (rating > 1). In the FA-Break session, a 3 min rest was introduced after mini-blocks 2, 3, or 4 when participants reported focused attention (rating = 1). No pauses were administered after block 1, as reduced focus at that point may have mostly reflected initial adjustment to the task rather than accumulated load, nor after block 5, which was always followed by a mandatory 10 min quiet resting period in all conditions.

After completion of all five blocks in each session, participants underwent the mandatory 10 min quiet rest and subsequently completed a final recall test without feedback, covering all 40 word pairs. Condition order (Break vs. No-Break in Study 1; MW-Break vs. FA-Break in Study 2) and stimulus lists were counterbalanced across participants.

To prevent participants from completing the two learning sessions consecutively, a Psychomotor Vigilance Task (PVT) was inserted between sessions. The PVT is a simple visual stimulus detection task originally developed to assess vigilance and to identify attentional lapses [50]. In the present study, the PVT was primarily used to limit carryover effects between the two experimental phases [51].

### 4.3. Statistical Procedure

All analyses were conducted using linear mixed-effects models (LMMs) to account for repeated measurements and inter-individual variability [52,53]. The consolidation index served as the dependent variable. The consolidation index was defined as delayed recall minus immediate recall performance (i.e., number of correctly recalled word pairs in cued immediate band delayed test). For the main analysis it was computed at the session level (i.e., delayed recall of the 40 pairs minus immediate recall summed across the five 8 word pair blocks). For MW intensity moderation analyses where breaks could be triggered only in blocks 2, 3, and 4, it was computed at the block level (e.g., delayed recall for the 8 word pairs belonging to encoding block 3 minus immediate recall for this same block 3), matching the block-wise assessment of MW intensity and pause triggering. Experimental condition was included as the predictor of interest. Recruitment, session, list, and condition order were included as covariates, selected a priori. A random intercept for Participant was included to account for inter-individual variability. *p*-values for fixed effects were obtained using Satterthwaite approximations (lmerTest package). Model assumptions (normality and homoscedasticity of residuals) were assessed through standard diagnostic plots [54,55].

To assess the robustness of the Condition effect with model specification, we conducted complementary analyses. First, we compared the full model to an otherwise identical model excluding Condition using a likelihood ratio test. Second, we fitted a reduced model including Condition only (without covariates) and compared the estimated effect of Condition across model specifications. Finally, to complement the frequentist analyses, we conducted a Bayesian analysis using the same model structure. Models were fitted using the brms package (version 2.23.0) with a Gaussian likelihood. Weakly informative priors were specified for fixed effects (normal(0, 5)), random-effect standard deviations (cauchy(0, 2)), and the residual standard deviation (cauchy(0, 2)) [56,57,58]. Posterior distributions were estimated using four Markov chain Monte Carlo chains with 4000 iterations each (1000 warm-up). Evidence for the effect of Condition was quantified using Bayes factors by comparing the full model to a null model excluding Condition. The interpretation of Bayes factors was based on the threshold framework introduced by Raftery [59]. All exploratory analyses were computed using the full model with the variables of interests.

## Data Availability

The original data presented in this study are openly available in the OSF repository at https://tinyurl.com/3rr8bakp (accessed on 3 April 2026).

## Abbreviations

The following abbreviations are used in this manuscript:

EEG	Electroencephalogram
FA	Focused attention
MW	Mind-wandering
LMM	Linear mixed-effects models
SWA	Slow-wave activity
SWS	Slow-wave sleep

## Appendix A. Sensitivity Analyses and Model Comparisons Study 1

### Appendix A.1. Sensitivity Analysis: Reduced Model Including Condition Only

As a sensitivity analysis, we fitted a reduced model including Condition only (without covariates) and compared the estimated effect of Condition to that obtained from the full model. This analysis was conducted to assess the robustness of the Condition effect to model specification rather than to formally compare model fit.

**Table A1:** Study 1. Estimated effect of Condition across model specifications.

Model	*β*	SE	*df*	*t*	*p*
Condition-only	2.06	0.83	33	2.47	0.019
Full	2.24	0.88	31	2.56	0.016

Note. Models were compared using likelihood ratio tests based on maximum likelihood estimation. Model Condition-only = condition + (1|participant) and Model Full = condition + recruitment + session + List + condition start + (1|participant).

### Appendix A.2. Contribution of Condition Beyond Covariates

To assess whether the effect of Condition contributed to model fit beyond the inclusion of covariates, we compared a full linear mixed-effects model including Condition, Recruitment, Session, List, and which condition they started with to an otherwise identical model excluding Condition. Models were fitted using maximum likelihood estimation and compared using a likelihood ratio.

**Table A2:** Study 1. Likelihood ratio test comparing the full model to a model without the condition.

Model	AIC	BIC	logLik	*χ* ^2^	*df*	*p*
Reduced	379.89	397.64	−181.95	-	-	-
Full	375.04	395.01	−178.52	6.85	1	0.009

Note. Models were compared using likelihood ratio tests based on maximum likelihood estimation. Model Reduced = session + List + condition start + (1|participant) and Model Full = condition + recruitment + session + List + condition start + (1|participant).

### Appendix A.3. Analyses Controlling for Number of Breaks

To examine whether the observed Condition effect in Study 1 could be explained by variability in the number of pauses, we conducted a within-condition analysis restricted to the MW-Break session.

**Table A3:** Study 1. Additional analyses controlling for number of breaks (NbBreaks) within the MW-Break condition.

Model	*β*	SE	*df*	*t*	*p*
LM	0.53	0.95	32	0.56	0.58

Note. Model LM = CI ~ NbBreaks (MW-Break condition only).

## Appendix B. Exploratory Post Hoc Contrasts Study 1

**Table A4:** Study 1. Post hoc contrasts between MW-Break and No-Break across levels of mind-wandering intensity. Contrast: MW-Break—No-Break.

MW Intensity	Estimate	SE	*df*	*t*	*p*
1	0.27	0.26	174	1.02	0.31
2	0.72	0.17	165	4.26	<0.01
3	1.17	1.17	175	4.59	<0.01
4	1.63	1.63	179	3.84	<0.01
5	2.08	2.08	180	3.42	<0.01

## Appendix C. Sensitivity Analyses and Model Comparisons Study 2

### Appendix C.1. Sensitivity Analysis: Reduced Model Including Condition-Only

As a sensitivity analysis, we fitted a reduced model including Condition-only (without covariates) and compared the estimated effect of Condition to that obtained from the full model. This analysis was conducted to assess the robustness of the Condition effect to model specification rather than to formally compare model fit.

**Table A5:** Study 2. Estimated effect of Condition across model specifications.

Model	*β*	SE	*df*	*t*	*p*
Condition-only	0.16	1.04	18	0.15	0.88
Full	0.17	0.91	16	0.19	0.85

Note. Models were compared using likelihood ratio tests based on maximum likelihood estimation. Model Condition-only = condition + (1|participant) and Model Full = condition + session + List + condition start + (1|participant).

### Appendix C.2. Contribution of Condition Beyond Covariates

To assess whether the effect of Condition contributed to model fit beyond the inclusion of covariates, we compared a full linear mixed-effects model including Condition, Session, List, and which condition they started with, to an otherwise identical model excluding Condition. As all participants came from the same source of recruitment, recruitement was not added as a covariable. Models were fitted using maximum likelihood estimation and compared using a likelihood ratio.

**Table A6:** Study 2. Likelihood ratio test comparing the full model to a model without the condition.

Model	AIC	BIC	logLik	*χ* ^2^	*df*	*p*
Reduced	202.4	212.23	−95.2	-	-	-
Full	204.36	215.82	−95.18	0.04	1	0.84

Note. Models were compared using likelihood ratio tests based on maximum likelihood estimation. Model Reduced = session + List + condition start + (1|participant) and Model Full = condition + session + List + condition start + (1|participant).

### Appendix C.3. Analyses Controlling for Number of Breaks

To address concerns regarding a potential variability in the number of pauses across participants, we conducted additional analyses in Study 2. First, we tested whether the number of breaks predicted consolidation performance when included as a covariate in the main model. Second, we tested whether the number of breaks differed between MW-Break and FA-Break conditions. Results are summarized in Table A7.

**Table A7:** Study 2. Additional analyses controlling for number of breaks.

Model	Predictor	*β*	SE	*df*	*t*	*p*
Covariable	Condition	0.23	0.78	14	0.29	0.77
Covariable	Number of breaks	1.44	0.69	20	2.09	0.049
NBreaks	Condition	−0.04	0.22	33	0.19	0.85

Note. Model Covariable = condition + Number of breaks+ recruitment + session + List + condition start + (1|participant). Model NBreaks had the number of breaks as a dependent variable (= condition + session + List + condition start + (1|participant)).

## Appendix D. Exploratory Post Hoc Contrasts Study 2

**Table A8:** Study 2. Post hoc contrasts between MW-Break and FA-Break across levels of mind-wandering intensity. Contrast: MW-Break—FA-Break.

MW Intensity	Estimate	SE	*df*	*t*	*p*
1	−1.16	0.28	92	−4.11	<0.01
2	0.27	0.24	91	1.13	0.26
3	1.7	0.47	93	3.64	<0.01
4	3.13	0.75	94	4.18	<0.01
5	4.56	1.04	94	4.38	<0.01

## Appendix E. Outlier Exclusion

Outliers were identified based on standardized residuals of the model and on standardized consolidation index score, where |z| > 3. One participant was identified as an outlier and excluded from the analyses (Figure A1). Although this participant reported discomfort during the experiment (headache due to light exposure and continuous white noise) and uncertainty about task instructions, these self-reports were not used as exclusion criteria and are reported here for transparency.
